# Bridging clinical and computational assessments: Standardizing kinematic reporting in modified mallet scale movements

**DOI:** 10.1371/journal.pone.0355605

**Published:** 2026-08-10

**Authors:** Ali İmran Yalçın, Oğuz Faik Seven, Merve Sarıipek, Semra Topuz, Mehmet Arif Adli, Tüzün Fırat

**Affiliations:** 1 Faculty of Physical Therapy and Rehabilitation, Hacettepe University, Ankara, Türkiye; 2 Movement Analysis Laboratory, Hacettepe University, Ankara, Türkiye; 3 Movement Analysis and Podiatry Application and Research Center, Hacettepe University, Ankara, Türkiye; 4 Mechanical Engineering Department, Gazi University, Ankara, Türkiye; 5 Department of Physical Therapy and Rehabilitation, Ordu University, Ordu, Türkiye; University of Perugia: Universita degli Studi di Perugia, ITALY

## Abstract

Accurate assessment of shoulder kinematics in Modified Mallet Scale (MMS) movements is essential for clinical evaluation and treatment planning in brachial plexus birth injury (BPBI). However, inconsistencies in reporting methods affect the reliability of kinematic data. This study aims to determine the optimal Euler sequence for reporting shoulder kinematics during MMS movements. Kinematic data from the unaffected arms of ten children with BPBI were collected using inertial measurement units and analyzed retrospectively. Three commonly adopted Euler sequences (ZXY, XZY, YXY) were analyzed. The occurrence of discontinuities, including gimbal lock, phase angle discontinuities, was quantified. Additionally, amplitude coherence was evaluated by comparing reported joint angles with clinically expected values. The ZXY sequence was the only method that exhibited gimbal lock, particularly in global abduction (M1), global external rotation (M2), and hand-to-neck (M3). XZY and YXY showed no gimbal lock; however, YXY showed two instances of phase angle discontinuities. Amplitude coherence analysis identified XZY and YXY as most suitable for M1 and M2, YXY for M3 and hand-to-mouth (M5), XZY for hand-to-back (M4), ZXY for hand-to-belly (M6). The method used to report shoulder kinematics has a major impact on the accuracy of MMS movement analysis. Our findings highlight the need for movement-specific, standardized reporting approaches that reduce discontinuities and maintain amplitude coherence. Establishing such standards is essential for enhancing clinical relevance and ensuring meaningful comparisons across studies, particularly in the evaluation of BPBI.

## Introduction

Modified Mallet Scale (MMS) has become one of the most widely utilized clinical tools for assessing shoulder movement in Brachial Plexus Birth Injury (BPBI), appreciated for its practicality, ease of use, quick administration and strong reliability [1,2]. It requires clinicians to score six specific shoulder movements on a 1–5 scale according to performance, reflecting shoulder range of motion. Among six movements, two primarily assess isolated shoulder elevation and rotation, while the other four involve functional movements resembling daily activities. These movements provide clinically relevant insights for both practitioners and researchers investigating upper extremity function [3–5]. MMS scores inform clinical decision-making and support consistent follow-up [6], including treatment planning and comparison of pre- and post-surgical outcomes [7–9]. Accordingly, ensuring scoring accuracy is critically important.

While MMS is a valuable tool for assessing shoulder movement in children with BPBI, its reliance on observational methods presents certain limitations [10,11]. Scoring may vary based on clinician experience and leading inconsistencies [12]. A recent study comparing MMS scores with kinematic assessments found discrepancies, with scores either under- or overestimated [13]. Therefore, to obtain more reliable data, it is recommended to use objective 3D motion capture systems, as movements occur in multiple planes [1,13–15]. Among available 3D capture modalities, inertial measurement unit (IMU)-based systems have been validated for upper-limb kinematic assessment in BPBI and related populations and are increasingly adopted in clinical movement analysis due to their portability and suitability for use with children in clinical settings [10,16].

Motion capture systems accurately measure segment positions and orientations, but converting these data into clinically meaningful joint angles requires careful modeling [17,18]. Clinicians often use Euler sequences aligned with joint coordinate systems to express motions as flexion–extension, adduction–abduction, and internal–external rotation [19]. However, these anatomical terms map imperfectly to the shoulder’s inherently three-dimensional motion, and the computed angles are sequence-dependent: changing the rotation order alters both magnitudes and interpretation [20], as exemplified by Codman’s paradox [21]. In practice, sequence-dependent singularities (gimbal lock: GL) and discontinuities (phase angle discontinuity: PAD) can degrade amplitude coherence (AC) within the angle time series, producing artificial amplitude inflation/attenuation and misleading phase flips across planes, thereby obscuring the underlying kinematics.

Accordingly, this study aims to identify an optimal and interpretable approach for reporting shoulder kinematics during MMS movements. We evaluate three commonly used Euler sequences (YXY, ZXY, XZY) implemented in established motion-capture workflows, assessing each by the frequency and severity of discontinuities and by its preservation of AC in the resulting angle waveforms. Given the large ranges of motion and task-specific coupling inherent to MMS, we hypothesize that the optimal Euler sequence may vary across movements. Our goal is to converge on a consistent reporting scheme for MMS shoulder kinematics that minimizes discontinuities, preserves AC, and enables clearer comparison and communication across clinical and research contexts.

## Materials and methods

### Participants

Upper body kinematic data (pelvis, thorax, head, and arms) were collected from 10 children with Narakas Type 2a BPBI using IMUs during clinical follow-ups. Based on previous studies suggesting that the unaffected arm can be used for comparison in kinematic analyses [22], data from the unaffected limbs of these children (6 girls, 4 boys; age: 6.6 ± 1.1 years) without upper extremity pathology were retrospectively analyzed. This group was chosen for their milder involvement and similar clinical presentation. No formal sample size calculation was conducted due to the study’s exploratory nature. The data from the motion capture system evaluation were retrospectively reviewed in accordance with the Institutional Review Board approval from Hacettepe University Physical Therapy and Rehabilitation Faculty Research Ethics Committee (Approvel Number-Date: FTREK 25/02–20.02.2025). This study used a subset of data collected in a previous project. The dataset was accessed on 01 April 2025. Authors had access to identifiable information in the original dataset, but no personal identifiers are included in this manuscript.

### Data collection

**Fig 1:**
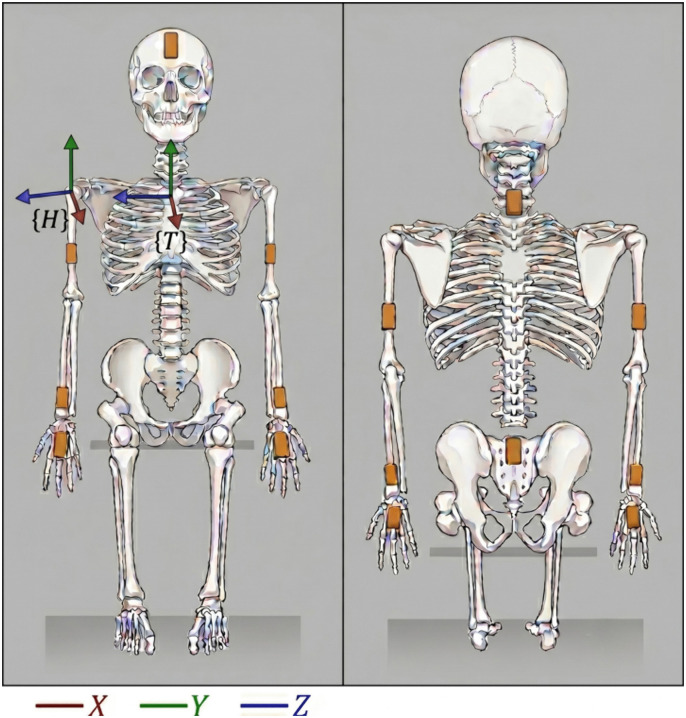
Illustration of IMU placement. The model is based on the OpenSim framework [24] and scaled to a representative participant’s proportions for visualization. The subject is depicted in a seated position during MMS assessments. This model serves solely to illustrate the Euler decomposition pipeline. H and T denote the local coordinate systems for the humerus and thorax, respectively.

**Fig 2:**
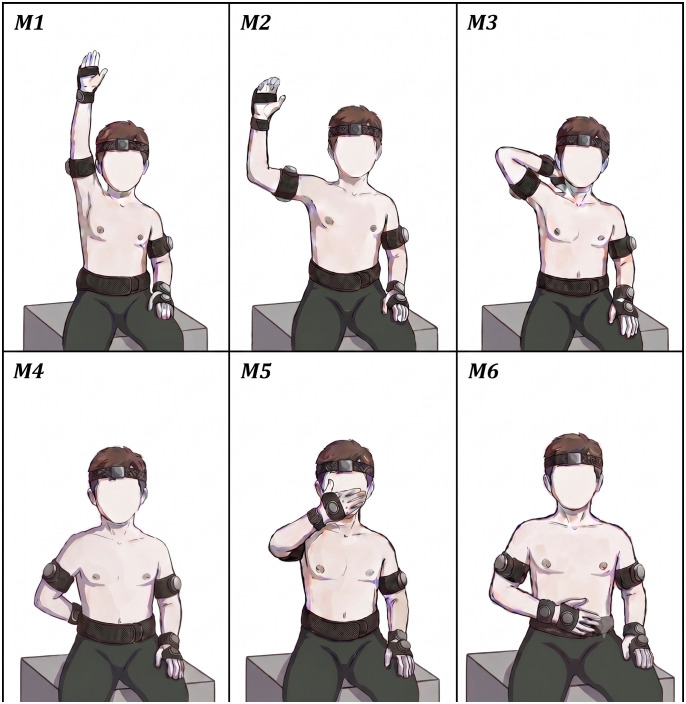
MMS movements. M1: global abduction, M2: global external rotation, M3: hand-to-neck, M4: hand-to-back, M5: hand-to-mouth, and M6: hand-to-belly (internal rotation).

Motion data were captured using nine IMUs: six on the upper arms, forearms, and hands, and three on the head, C7 and pelvis (Fig 1). Movella DOT sensors (Movella, Inc., Henderson, NV, USA [23]) recorded data at 60 Hz as children performed six MMS movements while seated without arm or back support, feet on the ground (Fig 2). To accommodate attention span limitations, each movement was repeated three times, with the most accurate trial selected for analysis. Trial accuracy was determined by visual inspection of concurrent video recordings by an experienced clinician (M.S.): the trial in which the child performed the target movement with the least compensatory trunk or multi-joint substitution was selected. Only IMU data from the trunk and unaffected arm was processed to calculate shoulder angles in this study.

### Data analysis

Initial processing (smoothing and sensor-to-segment alignment) was performed with a custom MATLAB script. The resulting data were then reformatted for OpenSim [25] compatibility. Finally, upper-body joint angles were obtained via inverse kinematics using a built-in full-body OpenSim model [24] (Fig 1) and the IMU Inverse Kinematics Tool [25]. To accurately compute joint angles for the specific rotation sequences evaluated (ZXY, XZY, and YXY), the shoulder joint definitions within the OpenSim model were modified for each sequence. Specifically, the original model’s ZXY joint coordinate system was redefined for the XZY and YXY sequences to ensure the mathematical decomposition aligned with the intended rotation order.

### Euler sequences

This study evaluates three common Euler sequences (YXY, ZXY, XZY). These three sequences were selected because they represent the Euler sequences most frequently reported in shoulder kinematics literature for MMS-analogous movements: ZXY and XZY are standard Cardan sequences used in OpenSim-based pipelines for anatomically aligned joint angles [24], while YXY is a proper Euler sequence recommended by International Society of Biomechanics (ISB) that uses clinically relevant coordinates [26]. Each method is computed using body-attached frames (Fig 1) based on ISB recommendations [26]. Euler sequences are usually assigned to the clinically relevant coordinates flexion/extension, adduction/abduction, internal/external rotation [27]. These sequences can also be used with an alternative coordinate set (plane of elevation, elevation angle, and axial rotation) where the first component of the Euler sequence defines the elevation angle, the second the elevation plane, and the last the axial rotation [17]. This shared framework enables direct comparison among the evaluated sequences.

### Evaluation metrics

The performance of each rotation sequence is evaluated based on two primary metrics:

#### Singularity and discontinuity occurrences.

**Fig 3:**
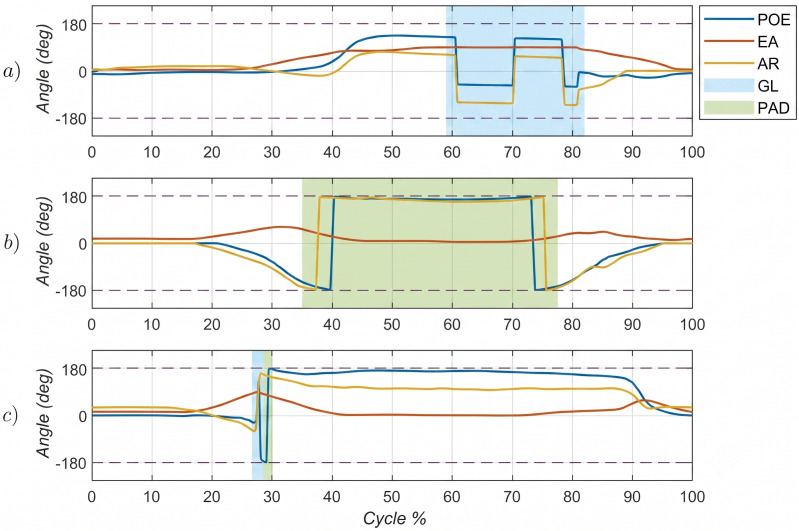
Representation of discontinuities. a) GL, b) PAD, c) GL and PAD together (POE: plane of elevation, EA: elevation angle, AR: axial rotation, GL: gimbal lock, and PAD: phase angle discontinuity).

In this study, both GL and PAD events are detected and number of occurrences are summarized as an evaluation metrics. GL arises in all Euler sequences when two rotation axes become collinear, effectively eliminating one degree of freedom and destabilizing the decomposition. In practice, GL occurs when the elevation angle approaches ±90° for the ZXY and XZY sequences, or 0°/180° for the YXY sequence (see Supplementary Material). At or near these configurations, the computed angles can exhibit abrupt, non-physiological excursions that artificially inflate or attenuate the underlying kinematics (Fig 3). Phase angle discontinuities (PADs) are identified when the absolute frame-to-frame difference in a computed joint angle reaches or exceeds 360° (Fig 3). Such discontinuities stem from angular wrap-around, particularly when trajectories cross the −180°/180° boundary, yielding sudden jumps unrelated to true motion [20].

#### Amplitude coherence.

When orientation data in kinematic analysis converted to joint angles via Euler sequences, the resulting values can deviate from clinical conventions. To preserve clinical relevance, joint-angle waveforms must be internally consistent and aligned with established interpretive standards. Although prior work reports kinematics for movements analogous to MMS tasks [28,29], heterogeneity in rotation sequences limits direct comparability. Therefore, to establish clinical interpretability, a priori expected clinical values and sign conventions were specified for each MMS movement (Table 1). The expected clinical ranges provided in Table 1 are defined based on the expert clinical opinion of our research team, reflecting our collective experience in evaluating shoulder function within clinical settings. These values serve as expert-derived reference intervals that characterize successful task performance as observed in our practice. These benchmarks allow for a more intuitive clinical interpretation of raw kinematic signals by providing a framework that aligns with our team’s established evaluation criteria. Furthermore, the signs for each movement were defined based on our clinical movement analysis protocols: positive values represent the intended functional motion (e.g., abduction, external rotation) relative to the standardized starting posture, while negative values denote motion in the opposite direction. This expert-driven framework facilitates the translation of 3D motion data into clinically actionable insights. Amplitude coherence is formally defined as the quantitative and directional agreement between the computed kinematics and the expected physiological ranges. To assess amplitude coherence, the mean peak joint angles achieved during each MMS movement were computed for all trials. A given Euler sequence was deemed coherent if its resulting mean peak angle successfully captured the correct anatomical direction and fell within the expected clinical bounds outlined in Table 1. All angular kinematics, computational thresholds, and expected physiological ranges in this study are calculated and reported strictly in degrees.

**Table 1:** Expected clinical values, adopted from [30].

Movements	M1	M2	M3	M4	M5	M6
**Plane of Elevation**	N/R	N/R	45∘−75∘	20∘ (N)−20∘	70∘−110∘	10∘ (N)−20∘
**Elevation Angle**	140∘−170∘	N/R	80∘−120∘	20∘−35∘	0∘−25∘	0∘−10∘
**Axial Rotation**	N/R	70∘−95∘ (ER)	40∘−80∘ (ER)	50∘−75∘ (IR)	5∘−30∘ (ER)	20∘−45∘ (IR)

All angular limits represent expected peak ranges and are presented in degrees. M1: global abduction, M2: global external rotation, M3: hand-to-neck, M4: hand-to-back, M5: hand-to-mouth, M6: hand-to-belly, ER: external rotation, IR: internal rotation, N: negative, and N/R: not required.

#### Decision Criteria for Optimal Sequence Selection.

To objectively determine the most appropriate rotation sequence for each MMS movement, we implemented a hierarchical selection framework based on the following three criteria:

1. Kinematic Stability (Priority 1): Sequences were primarily evaluated by the frequency of singularities (Gimbal Lock) and phase angle discontinuities (PAD). Methods exhibiting these artifacts were excluded from consideration for movements where these occurred.
2. Amplitude Coherence (Priority 2): For the remaining candidates, we compared the computed mean peak joint angles against the established expert clinical benchmarks (Table 1). A method was considered superior if its mean peak values aligned most closely with these clinical expectations.
3. Precision and Reliability (Priority 3): When multiple sequences demonstrated similar accuracy, the method exhibiting the lowest standard deviation in its angular output was selected, as this indicates greater consistency across participants.

## Results

The comparative analysis of joint angle reporting methods focused on evaluating the stability and coherence of the three primary Euler sequences (ZXY, XZY, and YXY) across six movements (M1-M6). Results immediately highlighted critical differences in singularity and discontinuity: ZXY was the only sequence having GL incidence, while XZY and YXY showed none. Specifically, ZXY showed GL in 4 out of 10 participants (40%) for M1, 4 (40%) for M2, and 2 (20%) for M3. PAD occurrences were limited to three (30% of participants) in M1 for ZXY and two (20% of participants) in M3 for YXY (Table 2).

**Table 2:** Comparison of discontinuity occurrences.

	M1	M2	M3	M4	M5	M6
**ZXY (%)**	40 (30)*	40	20	0	0	0
**XZY (%)**	0	0	0	0	0	0
**YXY (%)**	0	0	0 (20)*	0	0	0

Numbers indicate GL occurrences for Euler sequences. Values represent the number of affected participants out of ten (n = 10 trials total, one representative trial per participant per movement); percentages in parentheses indicate the proportion of participants affected. M1: global abduction, M2: global external rotation, M3: hand-to-neck, M4: hand-to-back, M5: hand-to-mouth, and M6: hand-to-belly. ()* indicates the number of PAD occurrences.

The analysis of joint angles derived from orientation data, using different reporting methods showed notable variability in mean joint angles and their standard deviations across movements. In the ZXY sequence, the plane of elevation showed moderate variation, while elevation angles exhibited substantial variability, particularly in M1 and M2. Axial rotation values were more consistent but still displayed noticeable differences across movements. For the XZY sequence, elevation angles remained stable across movements, while axial rotation and plane of elevation showed greater variability. The YXY sequence exhibited the greatest variability, especially in the plane of elevation and axial rotation, where differences between movements were substantial. Elevation angles in this sequence were relatively more consistent but still reflected some variation (Table 3).

**Table 3:** The mean and standard deviation of individual components evaluated at the peak of each motion.

Rotation Sequence	Rotation Axis	Movement	M1		M2		M3		M4		M5		M6	
**Mean(**^**o**^)	**SD(**^**o**^)	**Mean(**^**o**^)	**SD(**^**o**^)	**Mean(**^**o**^)	**SD(**^**o**^)	**Mean(**^**o**^)	**SD(**^**o**^)	**Mean(**^**o**^)	**SD(**^**o**^)	**Mean(**^**o**^)	**SD(**^**o**^)
**ZXY**	X	Plane of Elevation (Abduction)	15.1	8.7	66.8	12.8	30.1	13.0	24.9	23.2	−3.4	9.2	20.6	5.6
	Z	Elevation Angle (Flexion)	76.2	133.5	48.5	71.6	129.8	12.3	−37.8	22.3	50.3	12.9	0.7	14.9
	Y	Axial Rotation (Internal Rotation)	41.9	92.2	−25.9	69.2	44.7	21.8	47.8	25.5	51.3	14.7	40.4	14.7
**XZY**	Z	Plane of Elevation (Flexion)	30.8	21.0	18.8	14.4	40.3	11.2	−32.0	21.0	49.3	12.3	0.8	14.1
	X	Elevation Angle (Abduction)	161.3	8.9	86.1	12.7	138.0	15.7	28.2	29.4	−5.0	17.0	21.2	5.4
	Y	Axial Rotation (Internal Rotation)	−93.7	56.6	−73.4	9.3	−104.7	24.0	59.8	25.8	55.2	24.6	40.5	13.1
**YXY**	Y	Plane of Elevation	55.9	33.4	19.4	14.8	52.6	14.6	−49.2	36.6	93.9	12.0	1.4	28.5
	X	Elevation Angle	140.5	13.0	86.5	11.1	122.8	11.0	52.0	11.5	50.9	12.8	24.1	8.3
	Y	Axial Rotation	−42.7	36.1	−75.3	7.7	−67.9	19.1	103.3	30.0	−40.8	18.0	39.1	29.1

All angular outcomes are reported in degrees. M1: global abduction, M2: global external rotation, M3: hand-to-neck, M4: hand-to-back, M5: hand-to-mouth, M6: hand-to-belly, and SD: standard deviation.

**Fig 4:**
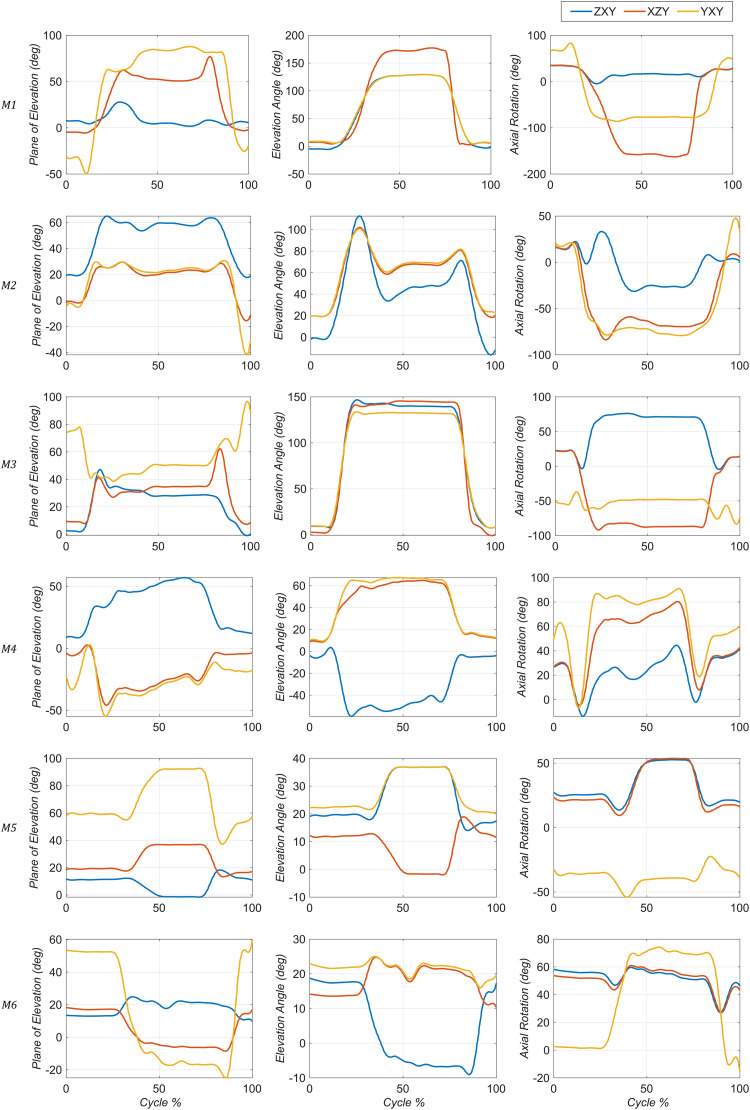
Representative Euler decomposition results for MMS movements. M1: global abduction, M2: global external rotation, M3: hand-to-neck, M4: hand-to-back, M5: hand-to-mouth, M6: hand-to-belly.

Fig 4 shows representative results for Euler sequences across MMS movements. While overall angle patterns were similar, variations occurred in amplitude and direction. In overhead movements (M1-M3), the plane of elevation and elevation angle remained consistent across Euler sequences, but axial rotation showed discrepancies. Specifically, in M1 and M3, ZXY reported internal rotation, while other methods resulted in external rotation. In M4 and M6, ZXY deviated from the others by reporting values in the opposite direction relative to XZY and YXY in the first two components. Similarly, in M5, ZXY yielded negative values for the plane of elevation compared to XZY and YXY, while YXY reported external rotation, in contrast to the internal rotation reported by the other methods.

After the performance of all reporting methods under consideration were analyzed in terms of singularity and discontinuity occurrences, and amplitude coherence, the final recommendations for each MMS movement are presented in Table 4.

**Table 4:** Recommended reporting methods for each MMS movement.

	M1	M2	M3	M4	M5	M6
**Recommended Reporting Methods**	XZY, YXY	XZY, YXY	YXY	XZY	YXY	ZXY

M1: global abduction, M2: global external rotation, M3: hand-to-neck, M4: hand-to-back, M5: hand-to-mouth, M6: hand-to-belly.

## Discussion

The purpose of this study was to identify the optimal rotation sequence for calculating shoulder kinematics during six MMS movements. To the authors’ knowledge, this is the first study to identify the most appropriate method for reporting shoulder kinematics in MMS movements. The findings highlight that the choice of rotation sequence significantly affects calculated joint angles, particularly during overhead elevation. While previous studies examined shoulder kinematics in MMS movements, few explicitly stated their preferred reporting methods [14,16], limiting clinical interpretability and complicating cross-study comparisons. The observed variability highlights the critical influence of rotation sequence, with some methods aligning more closely with expected clinical ranges. These findings emphasize the need for careful selection of reporting methods to improve kinematic data interpretability.

### Singularity and discontinuity occurrences

Among the Euler sequences analyzed, only the ZXY sequence exhibited GL occurrences. ZXY sequence experiences GL when its second component, the elevation angle, approaches 90°. Clinically, this corresponds to 90° of pure abduction [31]. All shoulder movements (M1-M6) occur predominantly in or around the frontal plane. Movements M1-M3 require an elevation angle of at least 90°, whereas M4-M6 can be performed with an elevation angle below 90°. Consequently, GL occurrences in M1-M3 and the absence of such occurrences in M4-M6 for the ZXY sequence are expected results. This aligns with findings from Senk and Cheze, which reported GL occurrences in the ZXY sequence during elevation in the scapular plane, anterior flexion, and circumduction, each involving shoulder elevation beyond 90° and resulting in GL in the decomposition [18].

In contrast, the XZY and YXY sequences exhibited no GL occurrences. The XZY sequence encounters GL when the shoulder is positioned near 90° of pure flexion [17], a position that none of the analyzed movements approached. Similarly, the YXY sequence experiences GL either when the shoulder is in the neutral position (0° elevation angle) or directly overhead (180° elevation angle) [32]. However, since all participants initiated and concluded movements with their hands positioned on their lap, the first GL-prone position of YXY was never reached. Although the second GL-prone position (180° elevation) is closely associated with M1, no GL occurrences were observed in M1 for YXY. The absence of GL in YXY during M1 was unexpected yet it can be explained with torso movement. Because the YXY decomposition defines the elevation angle relative to the thorax frame, lateral trunk lean or forward trunk flexion during maximal arm elevation reduces the humerothoracic elevation angle below the 180° singularity threshold. In the present dataset, children performing M1 showed variable trunk movement, and this compensatory torso displacement is sufficient to keep the computed elevation angle below the critical value at which GL would occur in YXY.

Among the evaluated sequences, XZY demonstrated superior performance by avoiding PAD occurrences, whereas ZXY and YXY exhibited PAD in specific movements. Unlike GL, PAD occurrences can be addressed through post-processing during range-of-motion calculations [33]. Consequently, we recommend that future researchers remain vigilant for PAD occurrences when employing the ZXY or YXY sequences, particularly in M1 and M3, respectively.

### Amplitude coherence

The evaluation of AC began with M1 which evaluates arm abduction, defined as elevation within the frontal plane. This movement may involve varying degrees of axial rotation based on natural tendencies, leading to higher standard deviations across reporting methods. The Mallet score for M1 considers only the elevation angle, excluding the plane of elevation and axial rotation from amplitude coherence assessment. For the elevation angle, ZXY reports a low mean amplitude accompanied by a high standard deviation (ZXY: mean 76.2°, SD 133.5°, versus expected 140°–170°). The mean amplitude is significantly below the expected value for the elevation angle, and the high standard deviation undermines coherence, suggesting that ZXY fails to capture the general movement pattern and may lead to erroneous conclusions. This elevated standard deviation is partially attributable to the occurrences of GL. In contrast, XZY and YXY report elevation angles that closely align with the expected amplitude values (XZY: mean 161.3°, SD 8.9°; YXY: mean 140.5°, SD 13.0°), making both methods suitable for analyzing M1.

Moving to M2 which primarily evaluates external shoulder rotation, independent of the elevation angle. Accurate scoring of M2 thus requires a coherent assessment of axial rotation. While ZXY report external rotation, amplitude measurements fall short of the expected values (ZXY: mean −25.9°, SD 69.2°, versus expected 70°–95° external rotation), rendering ZXY incoherent. Conversely, XZY and YXY provide axial rotation measurements that align with the expected amplitude values (XZY: mean −73.4°, SD 9.3°; YXY: mean −75.3°, SD 7.7°) and exhibit reasonable standard deviations. As such, these two methods are considered coherent for the evaluation of M2.

For the functional task M3, evaluating the ability to touch the hand to the neck, primarily achieved through elevation angle and axial rotation, with minor contributions from the plane of elevation. ZXY returned internal rotation values, rendering it incoherent for this movement, which requires external rotation. The axial rotation reported by XZY deviated significantly from the expected value and had a relatively higher standard deviation (mean −104.7°, SD 24.0°, versus expected 40°–80° external rotation), rendering it incoherent as well. Among all methods, YXY provided the most coherent amplitude values while maintaining acceptable standard deviations (mean −67.9°, SD 19.1°). Based on these findings, the YXY sequence is recommended for assessing M3.

A different challenge was presented by M4 which assesses shoulder mobility by evaluating the ability to touch the hand behind the back, primarily involving internal rotation [34]. Children who reach higher spinal segments receive higher MMS scores (on the 1–5 scale), while those who reach lower segments receive lower scores. Regarding the plane of elevation, none of the Euler sequences accurately reflect the expected range. ZXY comes closest but fails to capture the correct direction of motion for the elevation angle. For elevation angle alone, XZY is the only sequence to report values within the expected range (mean 28.2°, SD 29.4°, expected 20°–35°) and therefore recommended for M4.

M5 assesses the ability to bring hand to mouth, crucial for daily activities like eating and oral hygiene. While scoring M5 may seem straightforward, all three movement coordinates must be accurately assessed. Patients may compensate for low external rotation by increasing abduction (trumpet sign), reducing their score; in children with BPBI, this compensatory pattern is often related to the presence of internal rotation contracture [35,36]. Since external rotation is expected, YXY remains the only coherent method for axial rotation. The other sequences resulted in reversed rotation values within the expected range. However, YXY reports a significantly higher elevation angle than expected. Specifically, YXY yielded a mean elevation angle of 50.9° (SD 12.8°) for M5, compared to the expected range of 0°–25°. While YXY is strongly recommended for M5 due to its alignment with axial rotation expectations, researchers should interpret elevation angle data with caution.

The final movement assessed was M6, which evaluates the ability to touch the hand on the belly, primarily assessing internal rotation of the shoulder. It is an important metric especially for the realization of midline functions [37]. In terms of axial rotation, all sequences produced coherent results for M6. This is unsurprising, as M6 requires the smallest range of motion for humeral elevation among the movements assessed and is performed closest to the starting posture. However, in terms of plane of elevation and elevation angle, XZY and YXY reported values outside of the expected clinical range. As a result, only ZXY returned coherent values for all three components and recommended for M6.

Despite the novel insights provided by this kinematic analysis, the study is subject to some methodological limitations that warrant consideration. The descriptive nature and inherent structure of the analysis precluded the use of formal statistical inference, meaning the reported differences in kinematic coherence are qualitative rather than statistically verified. Because the objective was to evaluate reporting methodology rather than group-level kinematics, and because the same motion data were submitted to each rotation sequence simultaneously, formal statistical comparison of sequences would conflate methodological differences with inter-individual variability. Nonetheless, the mean and standard deviation values presented in Table 3 provide a quantitative basis for the amplitude coherence assessments. Future studies with larger samples could apply non-parametric or bootstrap-based inference to quantify uncertainty more formally. Furthermore, while joint angle data were collected from the unaffected limb of participants, the cohort was highly specific and had limited sample size, consisting solely of individuals with BPBI in the Narakas Type II classification. This specialized patient population inherently limits the generalizability of findings. Finally, the unaffected arm was deliberately chosen for this methodological study because it allows evaluation of Euler sequence performance on clinically normal, full-range shoulder kinematics without the confound of movement restrictions. Affected limbs in BPBI frequently exhibit reduced ranges and altered movement patterns that could limit or obscure the occurrence of discontinuities such as GL, which arise near specific angular thresholds. By using the unaffected limb we ensured that each rotation sequence was evaluated under biomechanically representative conditions where the full range of potentially problematic configurations could be encountered. It is acknowledged, however, that the Euler sequence recommendations derived here may require re-evaluation when applied to affected limbs with restricted motion or pronounced compensatory strategies; this constitutes an important direction for future work.

## Conclusion

Accurate characterization of shoulder motion is critical for daily-activity assessment and for informed decision-making in the management of BPBI and other upper-extremity disorders. Although kinematic analyses capture limb orientation, the clinical meaning of the angles depends on the reporting method. To align with clinical assessment and practice, shoulder kinematics should be reported in formats that clinicians can interpret readily; cross-method comparisons of angular values are discouraged because they undermine reliability. When singularities and discontinuities occur, they can artificially inflate/attenuate kinematic data. Extreme values should therefore exclude segments containing discontinuities. For continuous analyses, short discontinuity-affected intervals may be interpolated, but confidence declines as the gap lengthens; trials with extensive discontinuities should be omitted. Selecting a reporting method for MMS movements should prioritize amplitude coherence and minimize singularity and discontinuity occurrence, ideally using movement-specific recommendations. Adopting such tailored methods enhances the reliability and clinical relevance of shoulder kinematic assessments in both research and clinical settings. Although motivated by BPBI, these recommendations also apply to studies of movements that mirror MMS patterns.

## References

[1] Bae DS, Waters PM, Zurakowski D. Reliability of three classification systems measuring active motion in brachial plexus birth palsy. J Bone Joint Surg Am. 2003;85(9):1733–8. doi: 10.2106/00004623-200309000-00012 12954832

[2] Abzug JM, Chafetz RS, Gaughan JP, Ashworth S, Kozin SH. Shoulder function after medial approach and derotational humeral osteotomy in patients with brachial plexus birth palsy. J Pediatr Orthop. 2010;30(5):469–74. doi: 10.1097/BPO.0b013e3181df8604 20574265

[3] Tucker C, Bagley A, Wesdock K, Church C, Henley J, Masiello G. Kinematic modeling of the shoulder complex in tetraplegia. Top Spinal Cord Inj Rehabil. 2008;13(4):72–85. doi: 10.1310/sci1304-72

[4] Miller F. Physical examination and kinematic assessment of the upper extremity in cerebral palsy. Cerebral Palsy. Springer; 2020. pp. 1599–607.

[5] Seo NJ, Crocher V, Spaho E, Ewert CR, Fathi MF, Hur P, et al. Capturing upper limb gross motor categories using the Kinect(R) sensor. Am J Occup Ther. 2019;73(4):7304205090p1–p10. doi: 10.5014/ajot.2019.031682 31318673 PMC6638702

[6] Greenhill DA, Lukavsky R, Tomlinson-Hansen S, Kozin SH, Zlotolow DA. Relationships between 3 classification systems in brachial plexus birth palsy. J Pediatr Orthop. 2017;37(6):374–80. doi: 10.1097/BPO.0000000000000699 26633814

[7] Murabit A, Gnarra M, O’Grady K, Morhart M, Olson JL. Functional outcome after the Hoffer procedure. Plast Reconstr Surg. 2013;131(6):1300–6. doi: 10.1097/PRS.0b013e31828b7e0a 23714791

[8] Nath RK, Somasundaram C, Melcher SE, Bala M, Wentz MJ. Arm rotated medially with supination - the ARMS variant: description of its surgical correction. BMC Musculoskelet Disord. 2009;10:32. doi: 10.1186/1471-2474-10-32 19291305 PMC2664782

[9] Russo SA, Topley MT, Richardson RT, Richards JG, Chafetz RS, Rapp van Roden EA, et al. Assessment of the relationship between Brachial Plexus Profile activity short form scores and modified Mallet scores. J Hand Ther. 2022;35(1):51–7. doi: 10.1016/j.jht.2020.10.003 33308927

[10] Grip H, Källströmer A, Öhberg F. Validity and reliability of wearable motion sensors for clinical assessment of shoulder function in brachial plexus birth injury. Sensors (Basel). 2022;22(23):9557. doi: 10.3390/s22239557 36502259 PMC9736020

[11] Mahon J, Malone A, Kiernan D, Meldrum D. Kinematic differences between children with obstetric brachial plexus palsy and healthy controls while performing activities of daily living. Clin Biomech (Bristol). 2018;59:143–51. doi: 10.1016/j.clinbiomech.2018.09.004 30241094

[12] Delioğlu K, Unes S, Tuncdemir M, Ozal C, Bıyık KS, Uzumcugil A. Interrater reliability of face-to-face, tele- and video-based assessments with the modified Mallet classification in brachial plexus birth injuries. J Hand Surg Eur Vol. 2024;49(5):576–82. doi: 10.1177/17531934231196118 37684022

[13] Lovette M, Chafetz RS, Russo SA, Kozin SH, Zlotolow DA. Shoulder motion overestimated by Mallet scores. J Pediatr Orthop. 2024;44(10):e951–6. doi: 10.1097/BPO.0000000000002775 39034600

[14] Russo SA, Chafetz RS, Rodriguez LM, Roposh CM, Zlotolow DA, Kozin SH. Comparison of shoulder motion measurements by visual estimate, goniometer and motion capture. J Pediatr Orthop. 2022;42(8):443–50. doi: 10.1097/BPO.0000000000002212 35878417

[15] Russo SA, Kozin SH, Zlotolow DA, Nicholson KF, Richards JG. Motion necessary to achieve mallet internal rotation positions in children with brachial plexus birth palsy. J Pediatr Orthop. 2019;39(1):14–21. doi: 10.1097/BPO.0000000000001010 28834853

[16] Höglund G, Grip H, Öhberg F. The importance of inertial measurement unit placement in assessing upper limb motion. Med Eng Phys. 2021;92:1–9. doi: 10.1016/j.medengphy.2021.03.010 34167702

[17] Phadke V, Braman JP, LaPrade RF, Ludewig PM. Comparison of glenohumeral motion using different rotation sequences. J Biomech. 2011;44(4):700–5. doi: 10.1016/j.jbiomech.2010.10.042 21185023 PMC3042544

[18] Senk M, Chèze L. Rotation sequence as an important factor in shoulder kinematics. Clin Biomech (Bristol). 2006;21 Suppl 1:S3–8. doi: 10.1016/j.clinbiomech.2005.09.007 16274906

[19] Bonnefoy-Mazure A, Slawinski J, Riquet A, Lévèque J-M, Miller C, Chèze L. Rotation sequence is an important factor in shoulder kinematics. Application to the elite players’ flat serves. J Biomech. 2010;43(10):2022–5. doi: 10.1016/j.jbiomech.2010.03.028 20382388

[20] Creveaux T, Sevrez V, Dumas R, Chèze L, Rogowski I. Rotation sequence to report humerothoracic kinematics during 3D motion involving large horizontal component: application to the tennis forehand drive. Sports Biomech. 2018;17(1):131–41. doi: 10.1080/14763141.2016.1260765 28632057

[21] Cheng PL. Simulation of Codman’s paradox reveals a general law of motion. J Biomech. 2006;39(7):1201–7. doi: 10.1016/j.jbiomech.2005.03.017 15894325

[22] Wang JS, Petuskey K, Bagley AM, James MA, Rab G. The contralateral unimpaired arm as a control for upper extremity kinematic analysis in children with brachial plexus birth palsy. J Pediatr Orthop. 2007;27(6):709–11. doi: 10.1097/BPO.0b031e3180dca12a 17717476

[23] Alcala E, Voerman J, Konrath J, Vydhyanathan A. Xsens DOT wearable sensor platform white paper. White Paper. 2021. pp. 20.

[24] Rajagopal A, Dembia CL, DeMers MS, Delp DD, Hicks JL, Delp SL. Full-body musculoskeletal model for muscle-driven simulation of human gait. IEEE Trans Biomed Eng. 2016;63(10):2068–79. doi: 10.1109/TBME.2016.2586891 27392337 PMC5507211

[25] Delp SL, Anderson FC, Arnold AS, Loan P, Habib A, John CT, et al. OpenSim: open-source software to create and analyze dynamic simulations of movement. IEEE Trans Biomed Eng. 2007;54(11):1940–50. doi: 10.1109/TBME.2007.901024 18018689

[26] Wu G, van der Helm FCT, Veeger HEJD, Makhsous M, Van Roy P, Anglin C, et al. ISB recommendation on definitions of joint coordinate systems of various joints for the reporting of human joint motion--Part II: shoulder, elbow, wrist and hand. J Biomech. 2005;38(5):981–92. doi: 10.1016/j.jbiomech.2004.05.042 15844264

[27] Rab G, Petuskey K, Bagley A. A method for determination of upper extremity kinematics. Gait Posture. 2002;15(2):113–9. doi: 10.1016/s0966-6362(01)00155-2 11869904

[28] Aizawa J, Masuda T, Koyama T, Nakamaru K, Isozaki K, Okawa A, et al. Three-dimensional motion of the upper extremity joints during various activities of daily living. J Biomech. 2010;43(15):2915–22. doi: 10.1016/j.jbiomech.2010.07.006 20727523

[29] Magermans DJ, Chadwick EKJ, Veeger HEJ, van der Helm FCT. Requirements for upper extremity motions during activities of daily living. Clin Biomech (Bristol). 2005;20(6):591–9. doi: 10.1016/j.clinbiomech.2005.02.006 15890439

[30] Sarıipek M, Faik Seven O, İmran Yalçın A, Topuz S, Arif Adlı M, Fırat T. One sequence to show them all? Choosing the appropriate Euler sequence for shoulder kinematics in the Modified Mallet Scale. Gait Posture. 2025;121:226. doi: 10.1016/j.gaitpost.2025.07.242

[31] Amadi HO, Bull AMJ. A motion-decomposition approach to address gimbal lock in the 3-cylinder open chain mechanism description of a joint coordinate system at the glenohumeral joint. J Biomech. 2010;43(16):3232–6. doi: 10.1016/j.jbiomech.2010.07.034 20800843

[32] van Andel CJ, Wolterbeek N, Doorenbosch CAM, Veeger DHEJ, Harlaar J. Complete 3D kinematics of upper extremity functional tasks. Gait Posture. 2008;27(1):120–7. doi: 10.1016/j.gaitpost.2007.03.002 17459709

[33] Severin AC, Danielsen J. Rotation sequence and marker tracking method affects the humerothoracic kinematics of manual wheelchair propulsion. J Biomech. 2022;141:111212. doi: 10.1016/j.jbiomech.2022.111212 35780696

[34] Delioğlu K, Uzumcugil A, Öztürk E, Kerem Gunel M. Relative importance of factors affecting activity and upper extremity function in children with Narakas Group 2 brachial plexus birth palsy. J Hand Surg Eur Vol. 2021;46(3):239–46. doi: 10.1177/1753193420964768 33092449

[35] Noaman HH. Anterior shoulder release and tendon transfer as 1-stage procedure for treatment of internal rotation contracture deformity in obstetric brachial plexus injuries. Ann Plast Surg. 2013;71(5):510–8. doi: 10.1097/SAP.0b013e3182a1b02d 24126339

[36] van der Sluijs JA, van Ouwerkerk WJR, de Gast A, Nollet F, Winters H, Wuisman PIJM. Treatment of internal rotation contracture of the shoulder in obstetric brachial plexus lesions by subscapular tendon lengthening and open reduction: early results and complications. J Pediatr Orthop B. 2004;13(3):218–24. doi: 10.1097/00009957-200405000-00015 15083126

[37] Delioğlu K, Uzumcugil A, Öztürk E, Bıyık KS, Ozal C, Gunel MK. Cut-off values of internal rotation in the glenohumeral joint for functional tasks in children with brachial plexus birth injury. J Hand Surg Eur Vol. 2023;48(8):738–46. doi: 10.1177/17531934231154362 36788751

